# Supplementary material for: Acquisition of Escherichia coli carrying extended-spectrum ß-lactamase and carbapenemase genes by hospitalised children with severe acute malnutrition in Niger
Source: Nat Commun. 2025 Aug 1;16:6751. doi: 10.1038/s41467-025-61718-w (PMC12317043; doi:10.1038/s41467-025-61718-w)
Supplement: Supplementary file 1 — Supplementary Information [file 41467_2025_61718_MOESM1_ESM.pdf]

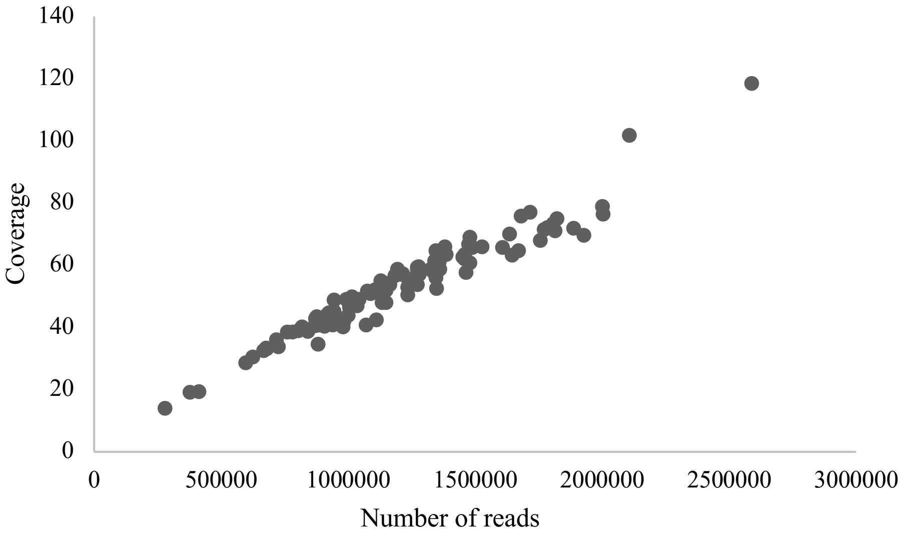

Supplementary Figure S1 – The coverage from trimmed paired end fastq files mapped to the corresponding *de novo* assembly. Only three bacterial isolates had coverage less than 30X and the median value was 53X.

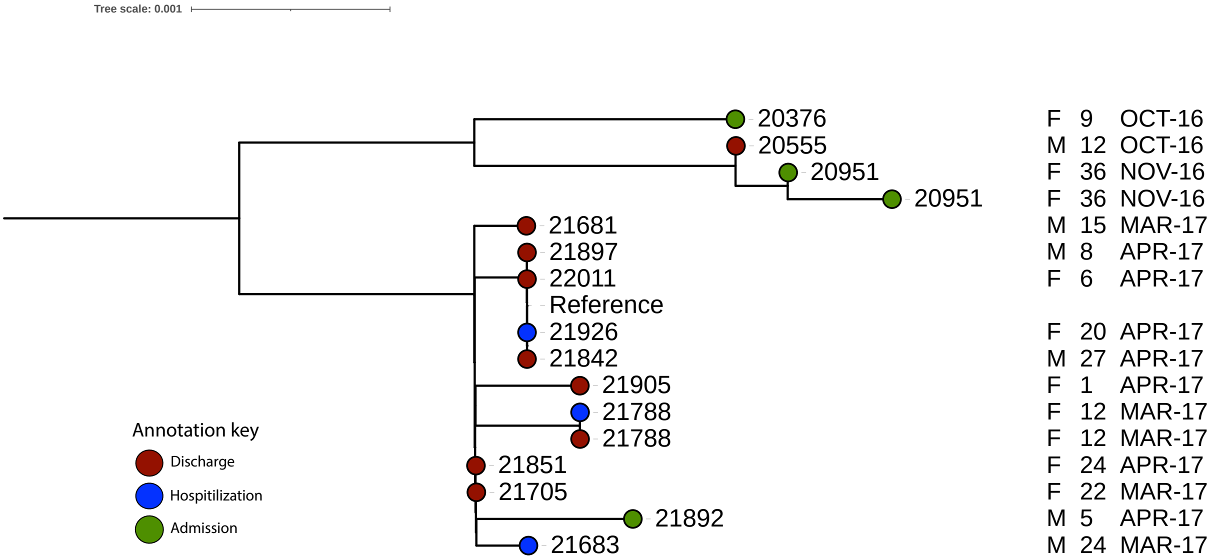

Supplementary Figure S2 - *E. coli* ST1284 collected from October 2016 through to April 2017. Four out of 16 were at the time of patient admission (green), three during hospitalisation (blue) and nine collected from sampling at patient discharge from the treatment facility (red) . The smaller clade contains the majority of the *E. coli* that were isolated upon admission, and, from samples collected between October and November 2016The larger clade contains samples collected between March and April 2017.

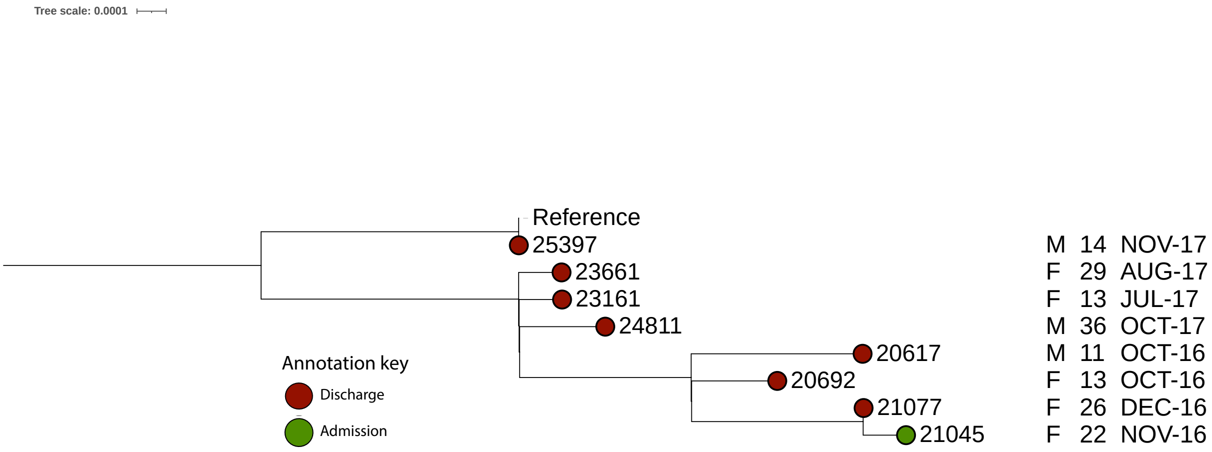

Supplementary Figure S3 – *E. coli* ST11025 *E. coli* collected from October 2016 through to November 2017. Seven out of eight isolates were collected from rectal swabs collected at patient discharge (red), and one was collected at the time of patient admission (green).

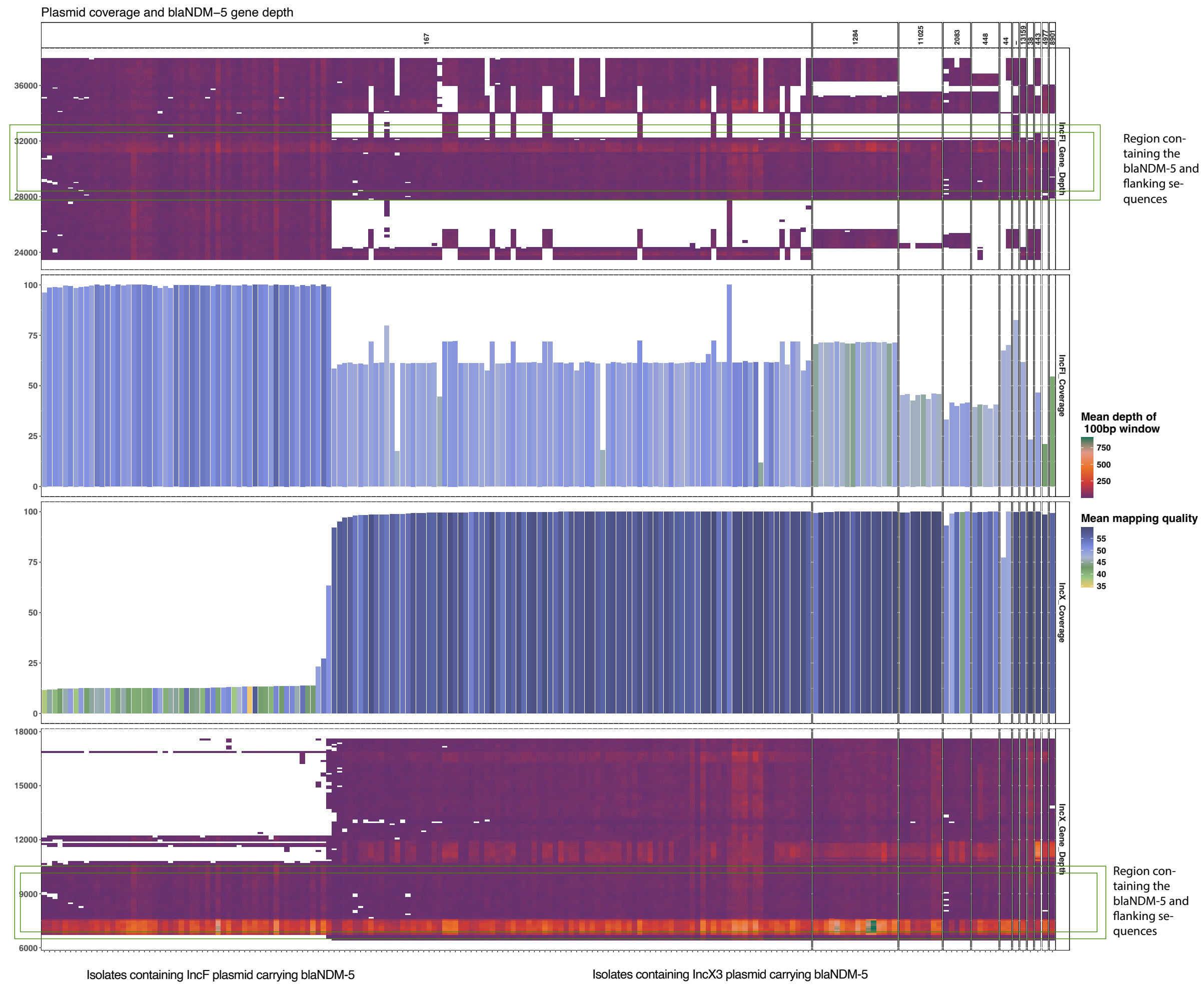

Supplementary Figure S4 – *E. coli* short read mapping to the IncF bla_{NDM-5} plasmid (pIncF-NDM5) and the IncX3 plasmid (pIncX3-NDM5-a). The ~3,000 bp sequence was common across the collection irrespective of plasmid carrying the bla_{NDM-5} gene, suggesting conserved flanking regions upstream and downstream the bla_{NDM-5} gene.
