# Supplementary material for: Acquisition of Escherichia coli carrying extended-spectrum ß-lactamase and carbapenemase genes by hospitalised children with severe acute malnutrition in Niger
Source: Nat Commun. 2025 Aug 1;16:6751. doi: 10.1038/s41467-025-61718-w (PMC12317043; doi:10.1038/s41467-025-61718-w)
Supplement: Supplementary file 2 — Reporting Summary [file 41467_2025_61718_MOESM2_ESM.pdf]

nature portfolio

Corresponding author(s): Kirsty Sands

Last updated by author(s): Jun 19, 2025

Reporting Summary

Nature Portfolio wishes to improve the reproducibility of the work that we publish. This form provides structure for consistency and transparency in reporting. For further information on Nature Portfolio policies, see our Editorial Policies and the Editorial Policy Checklist.

Statistics

For all statistical analyses, confirm that the following items are present in the figure legend, table legend, main text, or Methods section.

n/a	Confirmed	
☐	☒	The exact sample size (n) for each experimental group/condition, given as a discrete number and unit of measurement
☐	☒	A statement on whether measurements were taken from distinct samples or whether the same sample was measured repeatedly
☒	☐	The statistical test(s) used AND whether they are one- or two-sided<br>*Only common tests should be described solely by name; describe more complex techniques in the Methods section.*
☒	☐	A description of all covariates tested
☒	☐	A description of any assumptions or corrections, such as tests of normality and adjustment for multiple comparisons
☒	☐	A full description of the statistical parameters including central tendency (e.g. means) or other basic estimates (e.g. regression coefficient) AND variation (e.g. standard deviation) or associated estimates of uncertainty (e.g. confidence intervals)
☒	☐	For null hypothesis testing, the test statistic (e.g. F, t, r) with confidence intervals, effect sizes, degrees of freedom and P value noted<br>*Give P values as exact values whenever suitable.*
☒	☐	For Bayesian analysis, information on the choice of priors and Markov chain Monte Carlo settings
☒	☐	For hierarchical and complex designs, identification of the appropriate level for tests and full reporting of outcomes
☒	☐	Estimates of effect sizes (e.g. Cohen's d, Pearson's r), indicating how they were calculated

Our web collection on statistics for biologists contains articles on many of the points above.

Software and code

Policy information about availability of computer code

Data collection	No software was used in data collection. MSF team collected clinical/meta data from enrolled children. All data was either collected using pen and paper (very limited resources in the field), and later uploaded to Microsoft Excel/a computer, or directly entered into a spreadsheet depending on availability of a computer at the same.
Data analysis	nextflow (v20.10.100)<br>trimgalore (v0.6.4)<br>fastqc (v.0.11.8)<br>Shovill (v0.9.0).<br>Seqtk (v1.3)<br>Kraken (v2.0.8-beta)<br>Blast nt (v2.7.1)<br>bbtools/39.01<br>unicycler v.0.4.7<br>filtlong (v0.2.1)<br>Prokka (v1.14.5)<br>ABRicate (v1.0)<br>AMRfinderplus (v3.12.8)<br>PlasmidFinder (v1.0.)<br>mobsuite v3.1.8<br>mlst (v2.22.0).

bakta v1.9.3
dnaapler (v0.7.0)
PLDSB (v2024_05_31_v2)
Panaroo (v1.2.8)
IQtree v2
iToL (v6)
R studio (v4.2.0) and fuzzysim package, ggplot2, networkD3
unicycler, (v0.4.9)
snippy (v4.6.0)
Gubbins (v2.3.4)
snp-sites (v2.5.1)
Pairsnp (v.0.0.7).
flye (v2.9.4b)
dnaapler (v0.8.0)
nanoq (v0.10.0)
Samtools (v1.18)
clinker (v.0.0.31)
bakta (v1.9.4, database v5.1)

Geneious (2024 1.1)

For manuscripts utilizing custom algorithms or software that are central to the research but not yet described in published literature, software must be made available to editors and reviewers. We strongly encourage code deposition in a community repository (e.g. GitHub). See the Nature Portfolio guidelines for submitting code & software for further information.

Data

Policy information about availability of data

All manuscripts must include a data availability statement. This statement should provide the following information, where applicable:

- Accession codes, unique identifiers, or web links for publicly available datasets
- A description of any restrictions on data availability
- For clinical datasets or third party data, please ensure that the statement adheres to our policy

E. coli Illumina sequence reads, Illumina genomes, R9.4 and Illumina hybrid genomes used for variant calling, R10.4 whole genome assemblies, and R10.4 extracted plasmid sequences used for mapping are available in the NCBI repository under the project accession PRJNA1096457 (Source Data 5-6).

Research involving human participants, their data, or biological material

Policy information about studies with human participants or human data. See also policy information about sex, gender (identity/presentation), and sexual orientation and race, ethnicity and racism.

Reporting on sex and gender	Sex of the child was recorded as F for female or M for male.
Reporting on race, ethnicity, or other socially relevant groupings	This data was not collected or available for any analysis.
Population characteristics	Children from the community were admitted to the treatment facility based on severity of clinical symptoms. No further population characteristics were evaluated within this study.
Recruitment	A descriptive and longitudinal study was conducted between September 2016 and December 2017 in Madarounfa, Maradi Niger, at the Madarounfa Intensive Nutritional Rehabilitation Centre (CRENI) managed by the Niger Ministry of Public Health with support from MSF-OCP. Children admitted to the CRENI between 0 and 59 months of age who did not require immediate resuscitation on admission were enrolled. Blood and rectal samples were taken on admission.
Ethics oversight	The ethical evaluation of this study was carried out by the National Consultative Ethics Committee of Niger and the Committee for the Protection of Persons, Ile-de-France

Note that full information on the approval of the study protocol must also be provided in the manuscript.

Field-specific reporting

Please select the one below that is the best fit for your research. If you are not sure, read the appropriate sections before making your selection.

[x] Life sciences [ ] Behavioural & social sciences [ ] Ecological, evolutionary & environmental sciences

For a reference copy of the document with all sections, see nature.com/documents/nr-reporting-summary-flat.pdf

Life sciences study design

All studies must disclose on these points even when the disclosure is negative.

Sample size	Sample size was formed of all the children admitted to the treatment centre who consented to be part of the study. 3,004 rectal swabs were collected from 1,371 children.
Data exclusions	No. Children who did require immediate resuscitation upon admission were not enrolled.
Replication	All MIC data points were performed in duplicate with a third performed if the two datapoints were not equal
Randomization	No randomization required. All samples were processed.
Blinding	Not relevant to this study

Reporting for specific materials, systems and methods

We require information from authors about some types of materials, experimental systems and methods used in many studies. Here, indicate whether each material, system or method listed is relevant to your study. If you are not sure if a list item applies to your research, read the appropriate section before selecting a response.

Materials & experimental systems

n/a	Involved in the study	
☒	☐	Antibodies
☒	☐	Eukaryotic cell lines
☒	☐	Palaeontology and archaeology
☒	☐	Animals and other organisms
☒	☐	Clinical data
☒	☐	Dual use research of concern
☒	☐	Plants

Methods

n/a	Involved in the study	
☒	☐	ChIP-seq
☒	☐	Flow cytometry
☒	☐	MRI-based neuroimaging

Plants

Seed stocks	*Report on the source of all seed stocks or other plant material used. If applicable, state the seed stock centre and catalogue number. If plant specimens were collected from the field, describe the collection location, date and sampling procedures.*
Novel plant genotypes	*Describe the methods by which all novel plant genotypes were produced. This includes those generated by transgenic approaches, gene editing, chemical/radiation-based mutagenesis and hybridization. For transgenic lines, describe the transformation method, the number of independent lines analyzed and the generation upon which experiments were performed. For gene-edited lines, describe the editor used, the endogenous sequence targeted for editing, the targeting guide RNA sequence (if applicable) and how the editor was applied.*
Authentication	*Describe any authentication procedures for each seed stock used or novel genotype generated. Describe any experiments used to assess the effect of a mutation and, where applicable, how potential secondary effects (e.g. second site T-DNA insertions, mosiacism, off-target gene editing) were examined.*
